# Knowledge, Attitudes, and Practices Toward COVID-19 in a Saudi Arabian Population: A Cross-Sectional Study

**DOI:** 10.7759/cureus.8905

**Published:** 2020-06-29

**Authors:** Sarah K Almofada, Reem J Alherbisch, Nouf A Almuhraj, Bander N Almeshary, Bader Alrabiah, Abdulrahman Al Saffan, Mohammad A Baseer

**Affiliations:** 1 Dentistry, College of Dentistry, Riyadh Elm University, Riyadh, SAU; 2 Dentistry, College of Dentistry, King Saud University, Riyadh, SAU; 3 Family Medicine, King Fahad Medical City, Riyadh, SAU; 4 Preventive Dentistry, College of Dentistry, Riyadh Elm University, Riyadh, SAU

**Keywords:** coronavirus, covid-19, saudi arabia, population, pandemic, outbreak, acute respiratory infection, cross sectional studies, sars-cov-2, sars-cov-2 (severe acute respiratory syndrome coronavirus-2)

## Abstract

Background

Preventative measures are necessary to control a pandemic such as coronavirus disease 2019 (COVID-19), and different platforms to communicate guidelines have varying levels of effectiveness.

Objective

At the time of this study, there were no published studies investigating knowledge, attitudes, and practices related to COVID-19 within the Saudi population. Therefore, this study aims to address this gap in current knowledge and provide baseline data to the government and other health associations for preventive measurements for future outbreaks.

Methods

This study was conducted among the Saudi population using an online questionnaire. The questionnaire assessed the awareness of the severe acute respiratory syndrome coronavirus 2 (SARS-CoV-2) incubation period, the known symptoms of COVID-19, the mode of transmission of SARS-CoV-2, and infection control measures for prevention.

Results

The study included 6000 participants. Most respondents (84.93%) of the population reported awareness of the virus, 78.78% reported a belief that the virus leads to death, 89.47% reported a belief that 14 days is the incubation period, and 93.73% were aware of the possibility of asymptomatic carriers.

Conclusions

The Saudi population is aware of the attitudes and practices of prevention as well as the mode of transmission. The efforts by the Ministry of Health were effective in increasing awareness among the Saudi population toward COVID-19.

## Introduction

In December 2019, the World Health Organization (WHO) regional office in China was informed of cases of pneumonia of unknown cause detected in Wuhan City. In January 2020, Chinese authorities announced they had identified a new virus (severe acute respiratory syndrome coronavirus 2 (SARS-CoV-2)) that causes these cases [1]. On January 30, 2020, the WHO declared that the coronavirus disease (COVID-19) outbreak was the sixth public health emergency of international concern [2]. Saudi Arabia recorded the first confirmed case of COVID-19 on March 2, 2020. Globally, the World Health Organization has reported 533,416 laboratory-confirmed cases, 123,268 recoveries, and 24,110 deaths [3]. Several pneumonia cases of unknown etiology were linked by the investigations towards SARS-CoV-2 as the causative agent of the outbreak [4]. There are many routes of transmission of COVID-19. In Wuhan, China, the humans were affected via exposure to animals available in the Huanan Seafood Wholesale market. After that, human-to-human transmission has been occurring through droplets or direct contact (i.e., cough, sneeze) or indirect contact [5]. The transmission of the virus may occur via asymptotic patients due to the virus’s incubation period of one to 14 days [6].

Common clinical signs and symptoms of COVID-19 include fever, dry cough, dyspnea, fatigue, and standard or decreased white blood cells [4]. Uncommon symptoms are diarrhea, rhinorrhea, abdominal pain, anorexia, headache, and sore throat [7]. Older patients with cardiovascular disease, hypertension, and diabetes are more likely to have a respiratory failure or rapid progression to organ dysfunction or death in severe cases [4]. Critical worldwide cooperation and global communication efforts for preventing the further spread of the virus have occurred quickly. Specific prevention strategies, such as quarantine, self-isolation, and social distancing, were enforced, and if an individual had any contact with the virus, that person was taken to a specialized medical quarantine facility for the assessment and monitoring for symptoms. These national precautions likely helped prevent and reduce the spread of the virus [8]. The World Health Organization reference laboratories are providing confirmatory testing for COVID-19 such as non-culture-based diagnostic laboratory work and polymerase chain reaction (PCR) blood work, including hematology and clinical chemistry analysis on clinical specimens and respiratory specimens (such as nasopharyngeal and oropharyngeal swabs, sputum and/or endotracheal aspirate from patients who are suspected or confirmed infected with the virus) [9]. As of March 28, 2020, the WHO does not approve any treatment of COVID-19. The WHO specifies management for each situation of the infected individual, depending on the severity of the case [10]. Supportive therapy can be used such as antipyretic drugs to reduce fever, oxygen therapy, and patient quarantine. Because COVID-19 is a global issue, relevant clinical trials may take 12 to 24 months to conduct.

The aim of this study was to assess COVID-19 knowledge among the general population of Saudi Arabia.

## Materials and methods

We conducted this study using a 12-item, multiple-choice questionnaire on the platform SurveyMonkey.com. The study was done in the central, northern, eastern, western, and southern regions of Saudi Arabia. The survey was distributed in multiple universities, private and government institutions, websites, and via social media influencers (i.e., individuals who represent a local voice within these platforms and have a high number of followers) through Snapchat, Twitter, and WhatsApp platforms. Descriptive statistics of frequency distribution and percentages were calculated for the categorical variables (demographic variables and questionnaire items). The association between demographic variables and knowledge, attitude, and practices towards COVID-19 were assessed by applying the chi-square test and Fisher’s exact tests. A p-value of less than 0.05 was considered statistically significant. All the data were analyzed by using IBM SPSS Statistics for Windows, Version 25.0. (IBM Corp., Armonk, NY).

## Results

Of our total 6,000 responses, 59.9% of participants were male while 40.01% were female. Most (94.4%) were Saudi while 5.6% were non-Saudi. Table 1 presents a breakdown of our respondent's demographic information, including age, education levels, and geographic region. Half of our respondents were in central Saudi Arabia.

**Table 1:** Demographic variables of the study subjects

Variables	n	%
Male	3595	59.9%
Female	2405	40.1%
Nationality		
Saudi	5664	94.4%
Non-Saudi	336	5.6%
Respondent age		
18-24 years	1254	20.9%
25-34 years	2010	33.5%
35-44 years	1540	25.7%
45-54 years	447	7.4%
Older than 54 years	749	12.5%
Education		
Less than high school	235	3.9%
High school	1935	32.3%
Bachelor’s degree	3304	55.1%
Master’ degree and above	526	8.8%
Geographic region		
South	625	10.4%
North	552	9.2%
East	780	13.0%
West	1043	17.4%
Central	3000	50.0%

Most respondents (84.9%; n=5096) reported some awareness of the existence of COVID-19 while 15.1% (n=904) were not aware of the virus. Most respondents (78.8%, n=4,727) reported knowledge that COVID-19 could lead to death.

**Figure 1:**
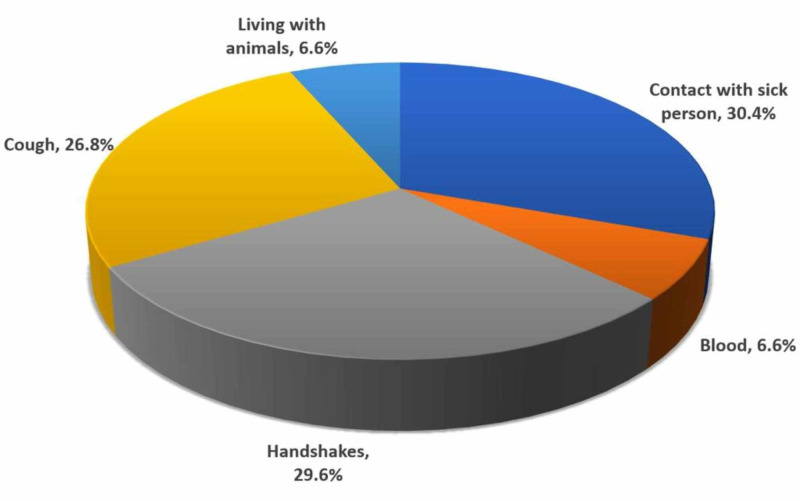
Ways of spread of COVID-19 infection (multiple responses)

Most respondents (30.4%; n=5945) reported an awareness that contact with a sick person could transmit the disease. A similar proportion (29.6%; n= 5783) reported awareness that the disease could transmit through handshaking. Figure 1 presents the distribution of knowledge of means of transmission.

Table 2 presents the association between demographic variables includes gender, nationality, age, education, region, and knowledge of the spread of COVID-19. 

**Table 2:** Association between demographic variables and knowledge of spread of COVID-19.

Variables		Contact with sick person			Blood			Handshakes			Coughs			Living with animals		
		No	Yes	p	No	Yes	p	No	Yes	p	No	Yes	p	No	Yes	p
Gender	Male	36.4	60.1	<0.001	61.4	54.7	<0.001	44.7	60.5	<0.001	64.3	59.3	0.008^*^	61.6	53.9	<0.001
	Female	63.6	39.9		38.6	45.3		55.3	39.5		35.7	40.7		38.4	46.1	
Nationality	Saudi	96.4	94.4	0.525	94.6	93.7	.204	91.2	94.5	0.039^*^	95.5	94.2	0.144	94.6	93.7	0.231
	Non-Saudi	3.6	5.6		5.4	6.3		8.8	5.5		4.5	5.8		5.4	6.3	
Age (years)	18 -24	38.2	20.7	0.011^*^	21.1	20.1	<0.001	25.3	20.7	<0.001	25.3	20.3	0.004^*^	21.5	18.7	<0.001
	25-34	25.5	33.6		33.0	35.4		19.4	34.0		31.2	33.8		33.0	35.3	
	35-44	12.7	25.8		25.1	27.6		22.1	25.8		22.8	26.1		24.9	28.4	
	45-54	9.1	7.4		7.0	9.2		14.3	7.2		6.4	7.6		7.2	8.2	
	>54	14.5	12.5		13.8	7.6		18.9	12.2		14.2	12.2		13.3	9.4	
Education	3.6	3.9	0.983	4.0	3.5	<0.001	7.8	3.8	0.018^*^	4.5	3.8	<0.001	3.9	3.9	<0.001
	High school	34.5		32.2	33.6		27.4	33.2		32.2	41.5		30.9	33.1		29.1
	Bachelor	52.7		55.1	55.3		54.4	49.8		55.3	48.6		56.0	55.6		53.1
	≥Master	9.1		8.8	7.1		14.7	9.2		8.7	5.5		9.2	7.3		14.0
Region	South	3.6	10.5	0.351	10.0	11.9	<0.001	7.8	10.5	0.176	8.8	10.7	0.015^*^	9.9	12.4	<0.001
	North	10.9	9.2		7.4	15.7		11.1	9.1		8.3	9.3		7.7	14.7	
	East	12.7	13.0		12.4	15.3		11.1	13.1		12.9	13.0		12.7	14.2	
	West	12.7	17.4		17.2	18.1		13.8	17.5		14.6	17.8		17.1	18.4	
	Central	60.0	49.9		53.1	38.9		56.2	49.8		55.5	49.2		52.7	40.3	

A significantly higher percentage of males as compared to the females were aware of the spread of COVID-19 through contact with a sick person ((60.1%) vs. (39.9%), (p<0.001)), blood ((54.7%) vs. (45.3%), (p<0.001)), handshake ((60.5% vs 39.5%, p=<0.001)), cough ((59.3%) vs. (40.7%)) and living with animals ((53.9%) vs. (46.1%), (p<0.001)). A significantly higher percentage of Saudi nationals as compared to non-Saudi nationals ((94.5% vs 5.5%, p=0.039)) were aware of the spread of COVID-19 through handshakes. However, the nationality, educational level, and region of the study participants did not demonstrate any significant difference in knowledge of the spread of COVID-19 through contact with a sick person (p>0.05). Study participants in various age groups were knowledgeable of the spread of COVID-19 through contact with the sick person (25-34 (33.6%), 35-44 (25.8%), 18 -24 (20.7%), >54 (12.5%) and 45-54 years (7.4%), (p=0.011)), blood (25-34 (35.4%),35-44(27.6%), 18-24 (20.1%), 45-54(9.2%) and >54 years (7.6%), (p<0.001)), handshakes (25-34 (34.0%), 35-44(25.8%), 18 -24 (20.7%), >54 (12.2%) and 45-54 years (7.2%), (p<0.001)), cough (25-34 (33.8%), 35-44 (26.1%), 18-24(20.3%), >54 (12.2%) and 45-54(7.6%), (p=0.004)), and living with animals (25-34 (35.3%), 35-44 (28.4%),18-24 (18.7%), >54 (9.4%) and 45-54 year (8.2%), (p=0.004)) (Table 2).

Study participants in different educational groups were aware of the spread of COVID-19 through blood (bachelor (54.4%), high school (27.4%), ≥master (14.7%) and <high school (3.5%), (p<0.001)), handshake (bachelor (55.3%), high school (32.2%), ≥master (8.7%), and <high school (3.8%), (p= 0.018)), cough (bachelor (56.0%), high school (30.9%), ≥master (9.2%), and <high school (3.8%), (p<0.001)), and living with animals (bachelor (53.1%), high school (29.1%), ≥ master (14.0%), and < high school (3.9%), (p<0.001)). Similarly, study subjects from various regions were aware of the spread of COVID-19 through blood (central (38.9%), west (18.1%), north (15.7%), east (15.3%), and south (11.9%), (p<0.001)), cough (central (49.2%), west (17.8%), east (13.0%), south (10.7%), and north (9.3%) regions (p<0.001)), and living animals (central (40.3%), west (18.4%), north (14.7%), east (14.2%), and south (12.4%) regions (p<0.001)) (Table 2).

**Figure 2:**
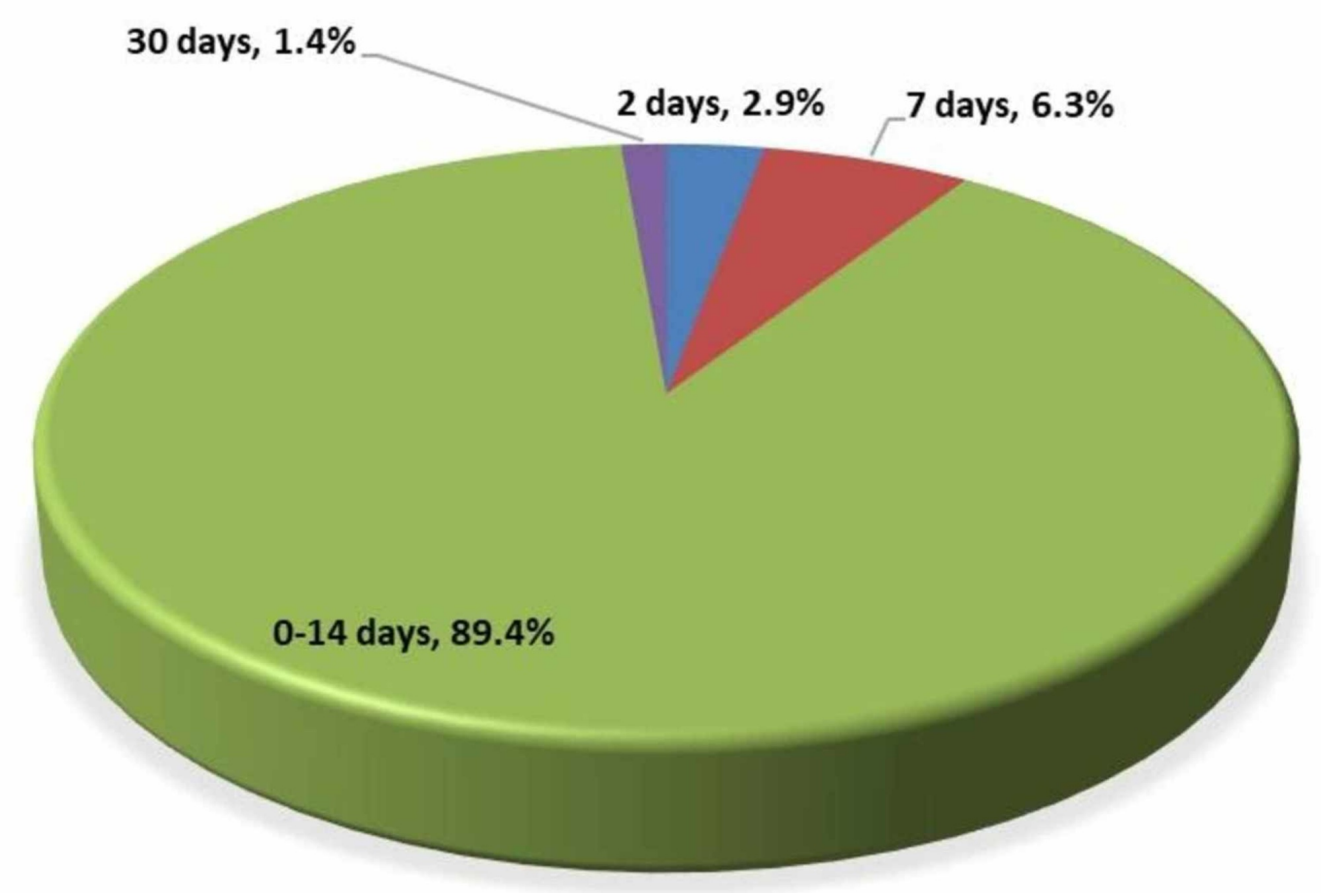
Knowledge of the incubation period of COVID-19

Figure 2 presents the responses on incubation duration. Most respondents (89.4%, n=5368) indicated that the incubation period is zero to 14 days. While (6.3%, n=375), (2.9%, n=176), and (1.4%, n=81) respondents mentioned that the incubation period was seven days, two days, and 30 days, respectively.

**Figure 3:**
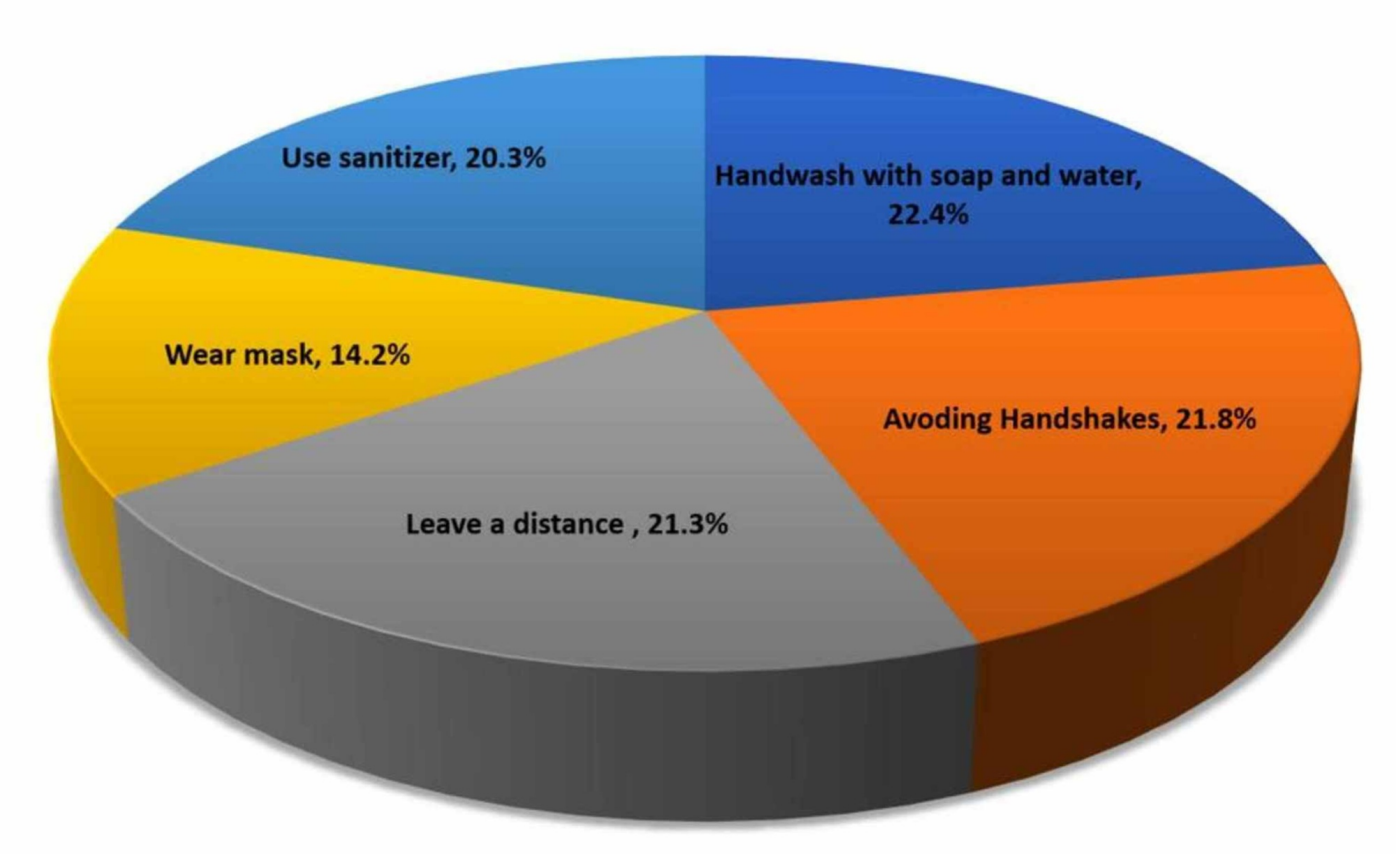
Avoiding the transfer of virus to others (multiple responses)

When asked about methods to avoid transmission of COVID-19, most respondents indicated washing hands with soap and water (22.4%; n=5955), avoiding shaking hands (21.8%,n=5801), keeping a distance from others (21.3%; n=5647), use of sanitizer (20.3%, n=5387), and wearing a mask (14.2%, n=3761), respectively. Figure 3 presents the proportion of respondents answers out of n=26551 responses for ways to avoid transmission of COVID-19.

Table 3 presents the association between demographic variables includes gender, nationality, age, education, region, and the knowledge of avoiding COVID-19.

**Table 3:** Association between demographic variables and knowledge of avoiding COVID-19

Variables		Hand wash with soap and water			Handshake			Leave distance			Wearing mask			Use of sanitizer		
		No	Yes	p	No	Yes	p	No	Yes	p	No	Yes	p	No	Yes	p
Gender	Male	62.2	59.9	0.751	34.7	60.8	<0.001	38.2	61.3	<0.001	53.2	63.9	<0.001	42.1	61.9	<0.001
	Female	37.8	40.1		65.3	39.2		61.8	38.7		46.8	36.1		57.9	38.1	
Nationality	Saudi	100.0	94.4	0.101	93.0	94.4	0.371	96.9	94.2	0.036^*^	95.2	93.9	0.044^*^	95.8	94.2	0.123
	Non-Saudi	0.0	5.6		7.0	5.6		3.1	5.8		4.8	6.1		4.2	5.8	
Age (years)	18-24	22.2	20.9	0.601	23.1	20.8	<0.001	25.8	20.6	<0.001	22.4	20.0	<0.001	15.3	21.5	<0.001
	25-34	33.3	33.5		24.1	33.8		21.2	34.3		32.2	34.3		21.0	34.9	
	35-44	24.4	25.7		23.6	25.7		23.5	25.8		22.4	27.6		24.6	25.8	
	45-54	2.2	7.5		16.6	7.1		12.2	7.2		7.4	7.5		13.4	6.8	
	>54	17.8	12.4		12.6	12.5		17.3	12.2		15.6	10.6		25.6	11.0	
Education	< High school	4.4	3.9	0.001^*^	7.0	3.8	0.042^*^	6.2	3.8	0.011^*^	3.9	3.9	0.006^*^	5.2	3.8	0.120
	High school	57.8	32.1		33.7	32.2		36.8	32.0		30.6	33.2		34.7	32.0	
	Bachelor	37.8	55.2		48.2	55.3		50.4	55.4		57.8	53.4		51.9	55.4	
	≥Master	0.0	8.8		11.1	8.7		6.5	8.9		7.7	9.4		8.2	8.8	
Region	South	4.4	10.5	0.471	13.1	10.3	<0.001	9.1	10.5	<0.001	6.1	13.0	<0.001	5.1	11.0	<0.001
	North	8.9	9.2		16.6	8.9		15.0	8.8		5.4	11.4		8.6	9.3	
	East	20.0	12.9		13.6	13.0		12.2	13.1		12.6	13.2		11.6	13.2	
	West	20.0	17.4		19.1	17.3		10.8	17.8		14.0	19.4		12.4	18.0	
	Central	46.7	50.0		37.7	50.4		53.0	49.8		61.9	42.9		62.3	48.6	

Knowledge of avoiding the spread of COVID-19 by leaving a distance was correctly answered by a higher percentage of males (61.3% vs 38.7%, p<0.001) and Saudi nationals (94.2% vs 5.8%, p=0.036) as compared to their counterparts. High proportions of study participants in the age category of 25-34 (34.3%), followed by 35-44(25.8%), 18-24(20.6%), >54 (12.2%), and 45-54(7.2%) categories showed knowledge of avoiding the spread of COVID-19 by leaving a distance (p<0.001). Similarly, leaving a distance to avoid spread differed significantly across bachelor (55.4%), high school (32.0%), ≥master (8.9%), and <high school (3.8%) educational categories (p=0.011). Nearly half of the study participants from the central region (49.8%) knew about avoiding the spread of COVID-19 by leaving a distance, followed by the west (17.8%), east (13.1%), south (10.5%), and north (8.8%) (p<0.001) (Table 3).

Knowledge of avoiding the spread of COVID-19 by wearing a mask was found in a higher percentage of males (63.9% vs 36.1%, p<0.001) and Saudi nationals (93.9% vs 6.1%, p=0.044) as compared to their counterparts. Similarly, different age categories (25-34 (34.3%), 35-44 (27.6%), 18-24 (20.0%), >54 (10.6%), and 45-54 (7.5%), (p<0.001)), educational categories (bachelor(55.4%), high school (32.0%), ≥ master (8.9%), and < high school (3.8%), (p=0.006)), and region (central (42.9%), west (19.4%), east (13.2%), south (13.0%), and north (11.4%), (p<0.001)) showed statistically significant differences towards the knowledge of avoiding the spread of COVID-19 by using a mask (Table 3).

A significantly higher percentage of males (61.9%) as compared to females (38.1%) showed the knowledge of avoiding the spread of COVID-19 by the use of sanitizer (p<0.001). Similarly, age categories (25-34 (34.9%), 35-44 (25.8%), 18-24 (21.5%), >54 (11.0%), and 45-54 (6.8%), (p<0.001)) and different regions (central (48.6%), west (18.0%), east (13.2%), south (11.0%) and north (9.3%), (p<0.001)) showed knowledge of avoiding the spread of COVID-19 by the use of sanitizer (Table 3).

Table 4 presents the association of demographic data with an awareness of asymptomatic transmission and susceptibility.

**Table 4:** Association between demographic variables and knowledge of the asymptomatic transmission and susceptibility of COVID-19

Variables		Asymptomatic carrier			Most susceptible COVID-19			
		Yes (%)	No (%)	p	Children (%)	Young (%)	Elderly (%)	p
Gender	Male	61.2	41.2	<0.001	52.1	75.3	58.9	<0.001
	Female	38.8	58.8		47.9	24.7	41.1	
Nationality	Saudi	94.4	94.9	.634	94.2	95.2	94.3	0.710
	Non-Saudi	5.6	5.1		5.8	4.8	5.7	
Age (years)	18 -24	20.9	21.3	<0.001	24.8	31.7	19.7	<0.001
	25-34	33.9	27.9		31.4	36.9	33.3	
	35-44	26.0	19.9		26.0	21.6	26.0	
	45-54	7.1	13.0		7.4	3.5	7.8	
	>54	12.1	17.8		10.3	6.2	13.2	
Education	< High school	3.7	7.2	<0.001	5.4	5.6	3.7	<0.001
	High school	31.9	37.2		38.8	41.3	31.1	
	Bachelor	55.4	50.0		49.6	48.5	55.9	
	≥Master	9.0	5.6		6.2	4.6	9.3	
Region	South	10.3	11.7	.157	12.8	15.8	9.8	<0.001
	North	9.1	10.4		16.9	7.7	9.0	
	East	13.0	12.8		12.4	12.9	13.0	
	West	17.7	12.8		15.7	19.9	17.2	
	Central	49.8	52.4		42.1	43.8	50.9	

A high proportion of the male (61.2%) participants as compared to the females (38.8%) was aware of the asymptomatic carriers of COVID-19. Similarly, significant difference in awareness was reported across different age groups 25-34 (33.9%), 35-44 (26.0%), 18-24 (20.9%), >54 (12.1%), and 45-54 (7.1%) (p<0.001) and educational categories bachelor (55.4%), high school (31.9%), ≥master (9.0%) and <high school (3.7%) (p<0.001). A high percentage of male study participants mentioned that the young age group (75.3%), followed by the elderly (58.9%) and children (52.1%) are most susceptible to COVID-19. While a high percentage of females said that children (47.9%) followed by the elderly (41.1%) and younger age groups (24.7%) were most susceptible to the COVID-19. Knowledge of susceptibility to COVID-19 infection demonstrated a significant difference between male and female study participants (p<0.001). Similarly, knowledge of susceptibility of children, young and elderly to COVID-19 differed significantly across various ages (p<0.001), education (p<0.001), and regional (p<0.001) categories of the study participants (Table 4).

## Discussion

According to our results, COVID-19 and its mitigation awareness among the Saudi Arabian population are high. Since the beginning of the pandemic, people sought relevant scientific information. Our questionnaire was based on the educational content provided by the Saudi Ministry of Health (MOH). The MOH used infographics and videos published on social media. Our survey sought to measure the transmission of important COVID-19 knowledge and guidance and whether platforms such as television, Twitter, and Snapchat were efficient for guideline publication. In addition, demographical variables (such as gender, nationality, age, education level, and geographic region) were recorded to discern the characteristic data of well-informed respondents. This assessment and correlation could help modify future efforts to best reach the groups that indicated low levels of awareness. The distribution was randomized around the regions of the Kingdom of Saudi Arabia, we noted a higher response from the central region of Saudi Arabia due to the region’s dense population and the social media influencers who helped in distributing the questionnaire. Many studies have examined the level of knowledge, attitude, and practices towards infectious diseases such as influenza, Middle East respiratory syndrome (MERS), and severe acute respiratory syndrome (SARS) [11-12], but no studies on COVID-19 in Saudi Arabia have been conducted. In a previous study that assessed the Middle East respiratory syndrome coronavirus (MERS-CoV), researchers reported that most participants had poor knowledge of the period of communicability (43.6%) [11]. The majority of participants in our study were aware of the current COVID-19 situation, answering “Yes” (84.93%) to the question “Did you hear about Coronavirus?” (84.93%). Our findings indicate an increase in community epidemic awareness from 2015 to 2020. Therefore, the efforts of the Saudi MOH to increase awareness and education have been effective. Our study was limited by its relatively sample size; Saudi Arabia has approximately 32 million people, and we collected 6000 responses. Also, we were limited by the current paucity of literature on COVID-19, as many studies are still ongoing.

## Conclusions

The data collected in this survey-based study can provide baseline data to the government and other health associations for preventive measurements in case of future outbreaks. Our results indicate that residents of Saudi Arabia have basic knowledge about the way of transmission, how to protect themselves from the virus, and the most chronological age groups are at risk of being affected by the virus. Collaborative efforts orchestrated by the MOH were efficient and effective, with a noticeable impact on public education towards prevention methods through multiple media platforms.
